# Rare Combined Congenital Pulmonary and Cardiovascular Malformations Revealed by Acute Hemoptysis

**DOI:** 10.1155/2024/1428495

**Published:** 2024-05-09

**Authors:** Dima Siblani, Ghassan Abi Chedid, Samir Challita, Sylvana el Zoghbi, Béatrice Le Bon Chami

**Affiliations:** ^1^Department of Pulmonary Medicine, Notre-Dame Maritime Hospital, Jbeil, Lebanon; ^2^Department of Interventional Radiology, Notre-Dame Maritime Hospital, Jbeil, Lebanon

## Abstract

**Background:**

Lung sequestration is a subtype of congenital lung malformations; it is infrequently diagnosed in adults and is a rare cause of hemoptysis. The typical management of symptomatic lung sequestration is usually surgical, though intra-arterial embolization is becoming an acceptable alternative. *Case Presentation*. We report a case of a 36-year-old female patient who presented for an acute onset of hemoptysis. CT chest showed an intralobar sequestration of the right lower lobe lung segment. In addition to the sequestration, the chest imaging also revealed a number of associated abnormalities including double superior vena cava communicating through a bridge, absence of brachiocephalic venous trunk, cardiac dextroposition, and agenesis of the right middle lobe. *Outcome and Discussion*. The transarterial embolization was selected for being mini-invasive and effective. It successfully controlled the bleed and led to complete regression of the sequestered lung on the follow-up CT chest several months later.

**Conclusion:**

Successful management and complete regression are possible with mini-invasive intra-arterial embolization of lung sequestration. Although it is not uncommon to have associated congenital cardiopulmonary abnormalities with lung sequestration, however the exceptional abnormalities described in this case have never been reported before.

## 1. Introduction

Congenital lung malformations (CLM) are uncommon lung developmental diseases seen in about 4 out of 10,000 live births. Diagnosis is typically antenatal or made later in infancy and is infrequent during adulthood. Bronchopulmonary sequestration (BPS) accounts for about 0.15-6.4% of all CLM and is defined as a nonfunctioning lung tissue that has aberrant blood supply and variable venous drainage and lacks communication with the tracheobronchial tree.

BPS remains asymptomatic in 15% of cases and is an unrecognized cause of recurrent pneumonia and hemoptysis. The classical management for symptomatic PBS is usually surgical; however, more interest is being given for minimally invasive embolization techniques.

Herein, we present the case of a 36-year-old female who presented for acute hemoptysis secondary to lung sequestration that was managed with transarterial embolization. The patient was also found to have a number of concomitant cardiopulmonary malformations.

## 2. The Case

A 36-year-old nonsmoker woman presented to our emergency department for hemoptysis. The bloody expectoration was about one tablespoon every two hours and occurred daily over the past two weeks. She had no fever, chest pain, dyspnea, or weight loss. No improvement was noted following a course of amoxicillin clavulanic acid and azithromycin. She had no history of prior hemoptysis or recurrent pneumonia and was not on blood thinner therapy. Her medical history included a surgical intervention for choanal atresia in childhood, minor thalassemia, and four miscarriages in first trimester of undetermined cause. Her family history was unremarkable.

**Figure 1:**
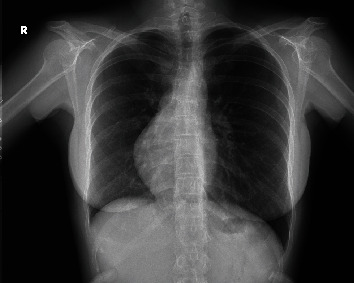
Chest X-ray showing the rightward shift of the heart.

**Figure 2:**
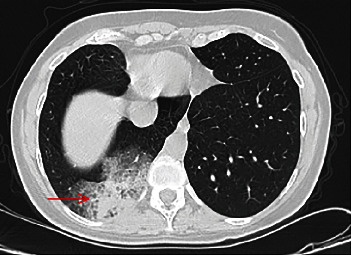
Axial view of the lower chest CT showing the consolidation and the ground glass opacity in the posterior segment of the right lower lung representing the sequestered lobe (red arrow).

**Figure 3:**
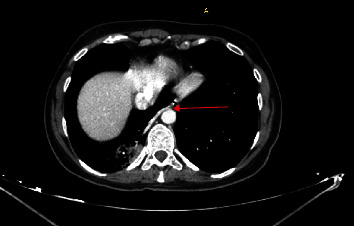
Axial view of the lower chest CT scan angiography showing the solitary aberrant artery (red arrow) originating from the left anterior border of the descending thoracic aorta and running rightward to supply the sequestered segment of the right lower lobe of the lung.

**Figure 4:**
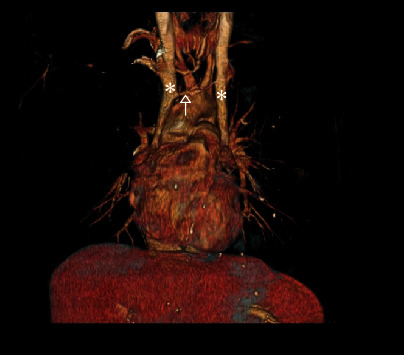
3D reconstruction view of the chest CT showing the two superior vena cava (white stars) with the communicating bridge passing anterior to the aortic arch (white arrow).

On admission, she was stable and saturated 98% on room air. Auscultation revealed some crackles at the right lung base. Laboratory investigations showed microcytic anemia (hemoglobin of 9 g/dL and a mean corpuscular volume of 57 fL). D-dimer and coagulation tests were within the normal range. Chest X-ray was relevant for a rightward shift of the heart (Figure 1). Computed tomography (CT) angiography of the chest showed an area of consolidation and ground glass opacity in the posterior segment of the RLL (Figure 2), vascularized by a systemic artery originating from the left anterior border of the descending aorta (Figure 3) suggestive of BPS. The sequestration drains into the right inferior pulmonary vein which supports the diagnosis of intralobular sequestration (ILS). Additionally, other congenital malformations were incidentally depicted: a double SVC with the absence of the brachiocephalic venous trunk and the presence of a communicating bridge between the two SVC anterior to the aortic arch (Figure 4). The left SVC runs posteriorly and inferiorly around the left ventricle and drains into the right atrium via the coronary sinus, while the right SVC drains directly into the right atrium. CT also showed dextroposition of the heart, pectus excavatum, and the absence of the middle lobar bronchus. Bronchoscopy was performed. It confirmed the absence of the RML and revealed pulsatile bleeding within the posterior segment of the RLL upon removal of blood clots. The hemorrhage ceased temporarily with local administration of cold saline and diluted adrenaline. The mucosa was pale. The rest of the bronchial anatomy was normal. Bronchoscopic cultures yielded negative results.

**Figure 5:**
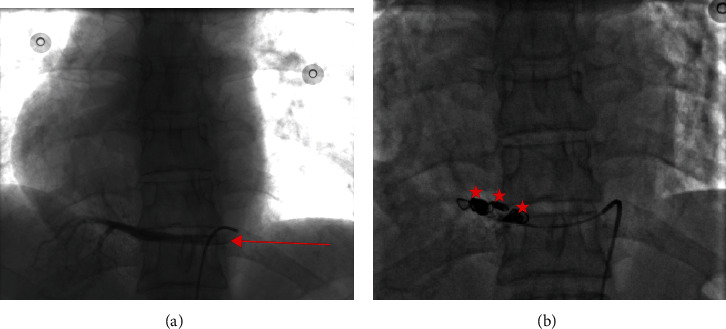
(a, b) Angiogram showing the aberrant artery arising from the left border of the descending aorta, before (red arrow) and after its complete occlusion with embolizing coils (red stars).

The patient's case was discussed in a multidisciplinary meeting including interventional radiologist, thoracic surgeon, and the pulmonary team, and an agreement was reached to adopt mini-invasive approach through transarterial embolization. The feeding artery of the sequestration originate from the thoracic aorta was successfully embolized using four 0.018-inch pushable coils (Balt, Montmorency, France) measuring 5 mm × 150 mm, 4 mm × 100 mm, and 2/3 mm × 80 mm, delivered by 2.4 Fr microcatheter (Progreat, Terumo, Japan) (Figures 5(a) and 5(b)). The aim of the procedure was to control the bleeding. No immediate complications were noted, the bleeding did not recur, and the patient was discharged home on day 2 postintervention.

Cardiac imaging confirmed the dextroposition. Transthoracic echocardiography, though technically challenging, raised suspicion on the right ventricular hypotrophy. However, cardiac MRI revealed a normal right ventricle, slight dilatation of the left ventricle (55 mm), and double SVC with the left one draining into the coronary sinus.

The surgical resection of the ILS lung following embolization was thoroughly deliberated within the multidisciplinary team, and consensus was in favor of watchful waiting strategy.

**Figure 6:**
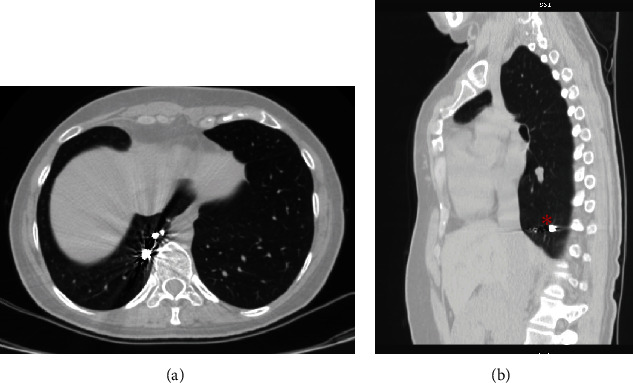
(a, b) Axial and sagittal views of the chest CT showing complete regression of the sequestered lobe with the embolization coils in place (red stars).

At the 10-month follow-up visit, the patient remained asymptomatic, and a follow-up CT scan showed complete regression of the ILS (Figures 6(a) and 6(b)).

## 3. Discussion

CPMs are divided into three categories, depending on whether the underlying anomaly is bronchopulmonary (lung agenesis, aplasia, or hypoplasia), vascular (usually a bronchial artery arising from the proximal descending aorta), or both (Scimitar syndrome, BPS, or other mixed lesions [1]. The CPMs described in our case belong to the second (agenesis) and the third category (BPS), and they were associated with additional congenital malformations, specifically cardiac dextroposition and double SVC. BPS accounts for 0.15 to 6.4% of all CPMs. It consists of a nonfunctional lung tissue that typically lacks communication with the tracheobronchial tree and receives its arterial blood supply from the systemic circulation (descending thoracic aorta in 76% of cases, upper abdominal aorta, coeliac or splenic arteries in 21% of cases, and occasionally the intercostal, subclavian, internal thoracic, or coronary arteries) [2]. In 16% of cases, multiple supplying vessels are identified. Venous drainage occurs through the pulmonary vein (in intralobar BPS) and less frequently into the systemic circulation (in extralobar BPS). Mixed pattern has been also reported [3]. Typically, intralobar BPS is not associated with other congenital malformations. However, concomitant diaphragmatic hernia, atrial septal defect, dextrocardia, and double SVC vein have been described [4]. One case report described an association with ipsilateral pulmonary hypoplasia, cardiac dextroversion, and a meandering right inferior pulmonary vein, but without duplication of the SVC [5]. BPS remains asymptomatic in 15% of cases. It is an unrecognized cause of recurrent pneumonia and hemoptysis. The latter occurs when there is airway communication, and although it is generally minor, it can become massive because of the elevated systemic pressures in the feeding artery. Other symptoms like cough, expectoration, fever, chest pain, and shortness of breath have been reported [2]. Atypical infections of BPS with tuberculosis, nocardia, and aspergillus have been described. Malignant transformations arising from BPS are rare occurrences and originate mostly within the sequestration or in direct proximity to it [6]. CT angiography of the chest is the preferred diagnostic modality allowing the visualization of the sequestered lung and its vascularization, as well as the detection of other thoracic abnormalities. The sequestration appears as consolidation, atelectasis, or solid lesion; and within it, cystic areas and air-fluid levels may be seen [1, 3].

Surgical resection has been the traditional management for BPS as it prevents the occurrence of associated complications. This strategy has become debatable for the asymptomatic BPS with the growing evidence of spontaneous regression [7]. More interest is being given to transcatheter embolization as a minimally invasive and effective alternative to surgery for the treatment of symptomatic BPS. This approach not only offers low rates of associated morbidities but also reduces the risk of intraoperative bleeding from the friable vascularization of the sequestrated tissue [4, 8].

In our patient's case, transarterial embolization was chosen in a multidisciplinary team meeting, for being an effective and a mini-invasive alternative to surgery. A follow-up chest CT performed 10 months later showed a complete regression of the ILS. A similar result has been also reported in the literature [7]. Regarding the potential risk of malignancy in patients with BPS, while there is no particular recommendation for patients treated with embolization, it seems prudent to keep vigilant surveillance.

## 4. Conclusion

The presented case describes a unique combination of congenital cardiovascular and pulmonary malformations. The diagnosis was made following an acute onset of hemoptysis originating from a pulmonary sequestration. A transarterial embolization controlled the bleeding and led, on the 10-month follow-up CT to complete regression of the sequestered lobe.

## Data Availability

All case-related data are accessible through the corresponding author (dima_siblani@hotmail.com) and can be shared upon request. No publicly archived datasets were used or generated during the study.
